# Supplementary material for: Chromothripsis during telomere crisis is independent of NHEJ, and consistent with a replicative origin
Source: Genome Res. 2019 May;29(5):737–49. doi: 10.1101/gr.240705.118 (PMC6499312; doi:10.1101/gr.240705.118)
Supplement: Supplemental Material [file supp_gr.240705.118_Supplemental_file_1.zip › contigs/annotated_contigs/DB53/contig.2.DB53_length_651_mean_cov_11.8832565284.docx]

**DB53_length_651_mean_cov_11.8832565284**

```
CTGGTGGCTAAATATTTTCTTTTGGCTTCTTGATCAATAAACAGCAGGAAGCTCTGGCTGTCCATTAGAATGCTTTCAGTAGCAAGACA
 >chr8:81115198-81115501 + E=1e-164
GAAAGCCCAACTCAAACTATTTAATTATAACAGAACTGGAAAGTTTAAAGGTAGAGCAGCCTTCAGAATGATTTGATTCAGTAGCTCTG

TAATAGCAAAGAACCTAGTTAGGGAGACAATCAAAATGTCTCACTTTTTCCTCTGGTCATACTGTTATAATACACACAACCATATAATG

ATACATCCTGTCTAGAACTAAGCCTACCA|ATAGATG|TGAGCCACTGCACCCTGCCAGGTGTGAGCCACTGCACCCCGCCAGTAATTC
                              >chr8:87682915-87683270 + E=7e-199
TCTTATTAACCCATCTGTACTGTCTTAACTTTGTTCTCTGTCTCACCACTCGTACCTTCCTGGATTACTCTCAAAATAAACTGTACGTA

AATCATTTTCTCAGGGTCTGCTTTTGGGAGATCCAAACTAAAACATCTGCAAAACACAGGTCCCCTCAGCACTTCTTTCTTCCCAGTAC

TTGGAGGGAGCAATGCTTACCAGTTTGTGGGCTGGCTTCGGGTGAGGAGAGATCTCCCTCTACCAACTTTTTCTTGTAGAGGGCTGTTC

TTTGACGCATTCTTTTCACCAGGTTGTGTA
```
